# Histology of gluten related disorders

**Published:** 2015

**Authors:** Michael N Marsh, Vincenzo Villanacci, Amitabh Srivastava

**Affiliations:** 1 *Department of Gastroenterology, Luton & Dunstable University Hospitals Trust, Luton, United Kingdom*; 2 *Wolfson College, University of Oxford, United Kingdom*; 3 *Institute of Pathology, Spedali Civili Brescia, Italy.*; 4 *Pathology department, Harvard Medical School, USA*

**Figure:**
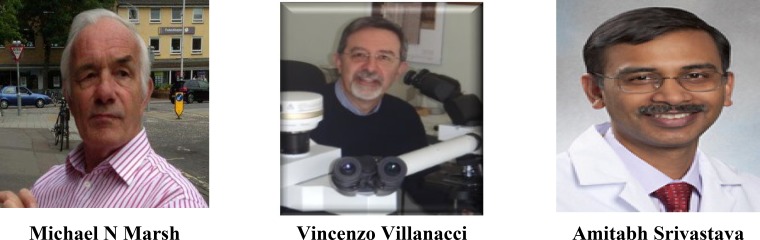


Gluten related disorders a range of inflammatory disorders of the small intestine characterized by malabsorption after ingestion of gluten in individuals with a certain genetic background. Clinical presentation can vary from full-blown malabsorption to subtle and atypical symptoms. Diagnosis currently relies on clinicopathologic studies including mucosal biopsy, serologic tests, and the effects of a diet free of gluten on the symptoms. Mucosal pathologic features are also variable, ranging from mild abnormalities, including intraepithelial lymphocytosis, to completely flat mucosa (1). Since there is no specific biomarkers for non-coeliac gluten sensitivity a combination of clinical and histology would play an improtant in identifying such individuals. Classification of mucosal pathology in gluten-sensitive enteropathy has been a subject of controversy among pathologists and needs to be revised according to the current understanding of the disease (2-4).

**Diagram:**
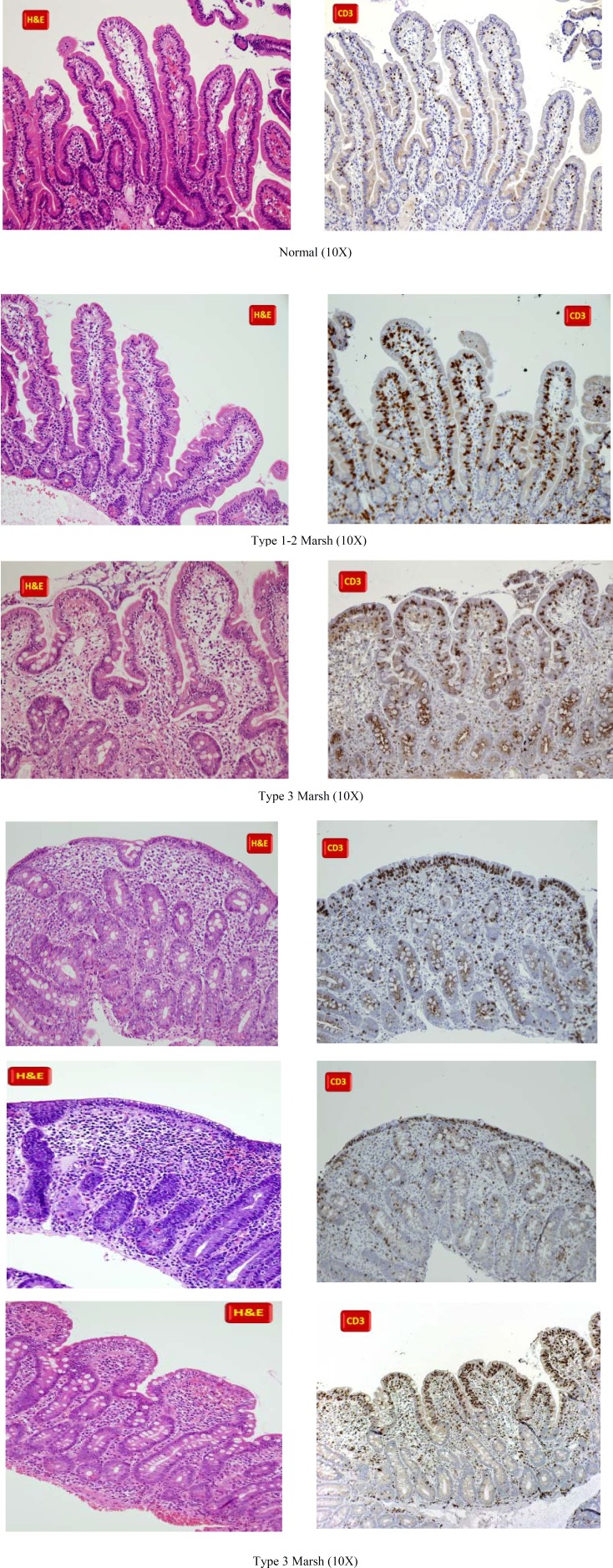
These data (Marsh MN et al, unpublished) illustrate the underlying immunopathologic features of celiac musosa as it becomes flat.

It is based on the computerised morphometric technique devised and employed in our laboratory, all measurements ultimately dependent on an invariant “comparator” of a constant test area of muscularis mucosae.

It refers not only to the crypts, but also the important changes occurring within the lamina propria: each stage has its characteristic changes, thus adding to the validity of the Marsh classification.

Overall, the inclusion of so many controlled numerical data in one diagram provides an almost dynamic view across the mucosa as it is becoming flat. We need further descriptive accounts, related to this underlying format, based on the evolutionary alterations in the production of inflammatory molecules and gene activations during the temporal development of the severe lesion.

This micrograph shows a Marsh II lesion, with well-preserved villi, and hypertrophied crypts. The epithelium is heavily infiltrated with small, non-mitotic lymphocytes. This specimen actually came from a first degree relative, and was one of the original findings which lead to the formulation of the Marsh Classification.

**Celiac disease**

**Figure:**
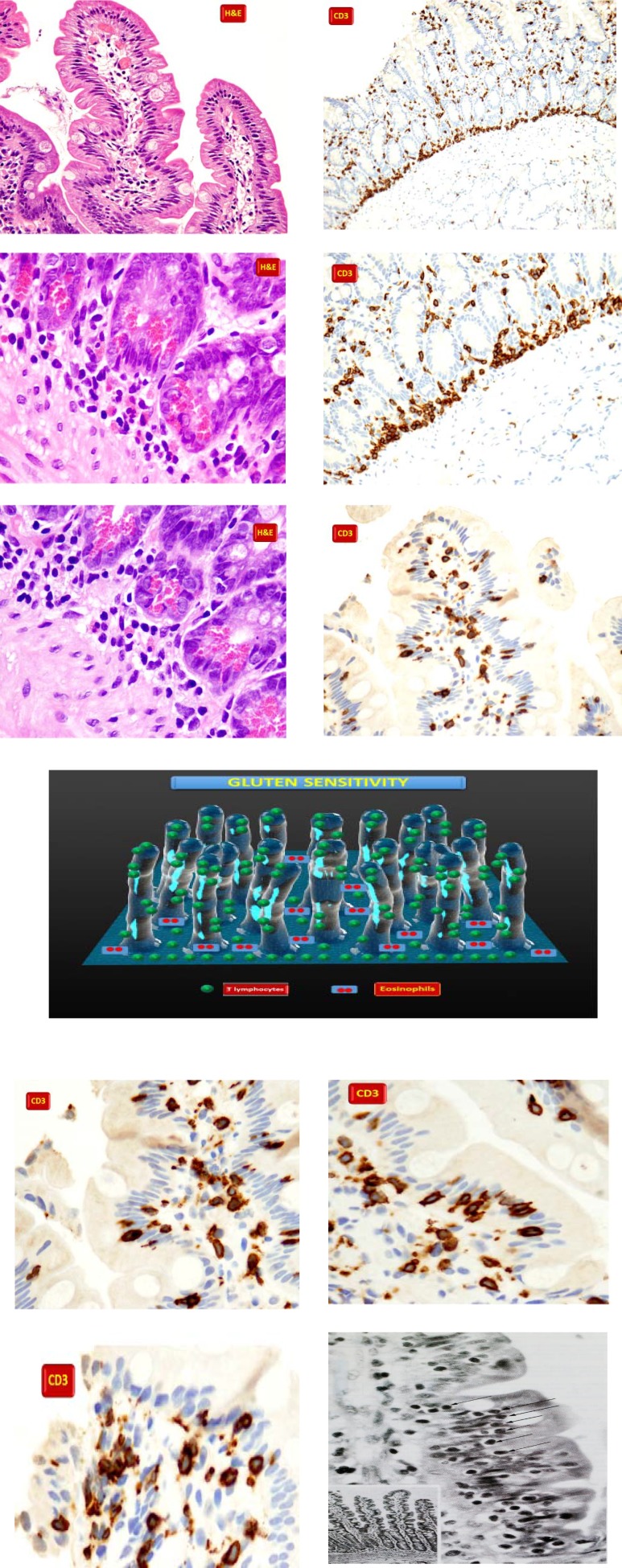


**Non Celiac Gluten Sensitivity**

**Figure:**
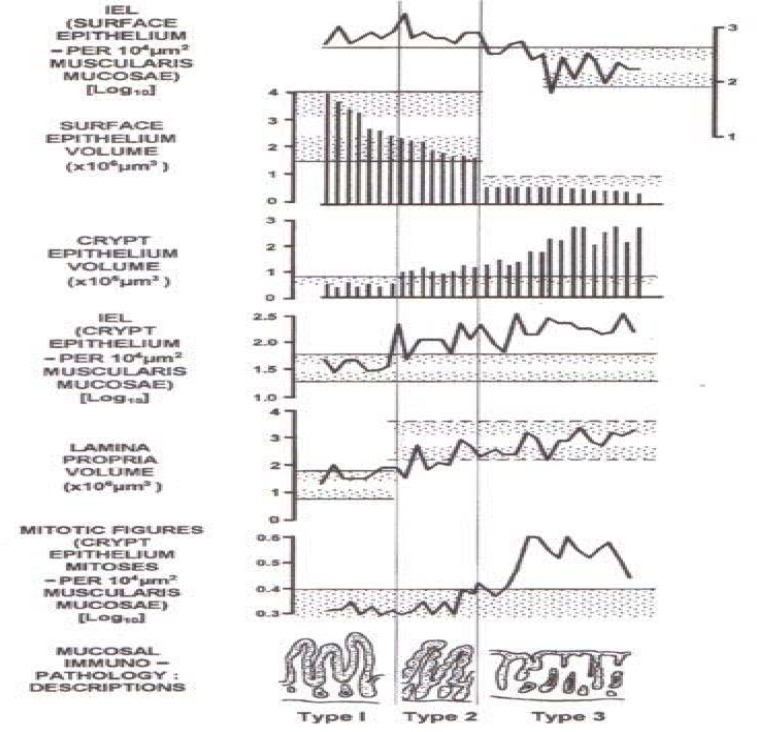


**Figure.**
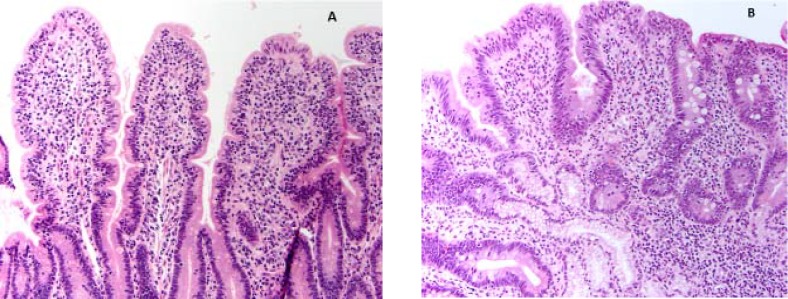
A variety of diseases can mimic celiac disease on histology. Tropical sprue (A) may show marked intraepithelial lymphocytosis (IEL). The lack of significant villous atrophy in the presence of significantly increased IELs should raise suspicion for tropical sprue. Total or subtotal villous atrophy with increased IELs mimicking celiac disease can also be seen in patients with immune deficiency disorders, as seen here in a patient with common variable immunodeficiency (B).
